# Appropriate Combination of Nalbuphine Combined With Esketamine for Weakly Opioid Anesthesia in Postlaparoscopic Cholecystectomy Analgesia: A Randomized Controlled Trial

**DOI:** 10.1155/prm/5086518

**Published:** 2026-03-03

**Authors:** Mengyun Zhang, Hao Wu, Han Zheng, Jing Zhang, Jiaxuan Wang, Hong Luo, Heng Yang

**Affiliations:** ^1^ Department of Anaesthesiology, The Third Affiliated Hospital of Anhui Medical University, The First People’s Hospital of Hefei, Hefei, 230061, Anhui, China, ciss.org.cn; ^2^ Department of Anaesthesiology, Fuyang Women’s and Children’s Hospital, Fuyang, 236031, Anhui, China

**Keywords:** esketamine, laparoscopic cholecystectomy, nalbuphine, patient-controlled intravenous analgesia

## Abstract

**Context:**

Laparoscopic cholecystectomy (LC) often leads to abdominal wall incision pain, visceral pain, and shoulder and neck pain after surgery. Nalbuphine provides strong sedative and analgesic effects with minimal risk of adverse reactions. Esketamine, with its higher receptor affinity, significantly alleviates both visceral and somatic pain.

**Objectives:**

Our objective was to establish the optimal dosage of nalbuphine with esketamine for patient‐controlled intravenous analgesia (PCIA) following LC, enhancing postoperative pain management for these patients.

**Methods:**

This research employs a single‐blind, randomized controlled trial methodology. Surgical patients were randomly assigned to three groups based on postoperative PCIA drug combinations: Group N received nalbuphine 2 mg·kg^−1^ with 100 mL of 0.9% sodium chloride solution; Group NE_1_ received esketamine 0.5 mg·kg^−1^, nalbuphine 2 mg·kg^−1^, and 100 mL of 0.9% sodium chloride solution; Group NE_2_ received esketamine 1 mg·kg^−1^, nalbuphine 2 mg·kg^−1^, and 100 mL of 0.9% sodium chloride solution. The study observed VAS scores at rest and during activity, Ramsay sedation scores, and MoCA scores at 6, 12, 24, and 48 h postsurgery, along with the total and effective PCIA pressing times across the three groups. Adverse reactions and the frequency of rescue analgesia within 48 h postsurgery were monitored.

**Results:**

Compared to the N group, VAS scores and resting pain scores were substantially lower in the NE_1_ and NE_2_ groups at 6 h, 12 h, and 24 h postsurgery (*p* < 0.05). Compared to Group N, Groups NE_1_ and NE_2_ had reduced postactivity VAS scores at 48 h postsurgery (*p* < 0.05). Compared to Group NE_1_, Group NE_2_ had reduced VAS scores at 12 h and 24 h following surgery (*p* < 0.05). Compared with Group N, the Ramsay sedation scores were higher in Group NE_1_ and Group NE_2_ at 6 h, 12 h, and 24 h following surgery (*p* < 0.05). Compared with Group NE_1_, Group NE_2_ had higher Ramsay sedation scores at 6 h and 12 h postoperatively (*p* < 0.05). The total number of analgesic pump presses and the number of effective presses in the NE_1_ and NE_2_ groups were substantially lower than those in the N group (*p* < 0.05). Compared with the NE_1_ group, the total number of presses and the number of effective presses in the NE_2_ group were reduced at 24 h and 48 h postoperatively (*p* < 0.05). Compared with Group N, the cognitive scores on the MoCA scale were markedly increased in Group NE_1_ and Group NE_2_ at 6 h, 12 h, and 24 h postoperatively (*p*  < 0.05). The incidence of vomiting was substantially lower in the NE_1_ and NE_2_ groups than in the N group at 48 h postoperatively (*p* < 0.05). The rate of drowsiness was markedly increased in the NE_2_ group than in the N and NE_1_ groups during the 48 h postoperative period (*p* < 0.05).

**Conclusion:**

The combination of nalbuphine (2 mg kg^−1^) and esketamine (0.5 mg kg^−1^) effectively relieves postsurgical pain, reduces the incidence of postoperative PCIA use, and lowers the incidence of adverse events after LC.

**Trial Registration:**

ClinicalTrials.gov identifier: ChiCTR2400088373

## 1. Introduction

Laparoscopic cholecystectomy (LC) is one of the most commonly conducted minimally invasive surgeries. The mechanisms underlying postoperative pain are complex, diverse, and different from those of traditional open surgery. In addition to the pain caused by abdominal wall incision, high intra‐abdominal pressure, peritoneal dilatation due to carbon dioxide perfusion, and diaphragmatic irritation can lead to visceral pain and shoulder and neck pain. Studies have shown that approximately 65% of patients experience moderate pain and 23% experience severe pain within 24 h after LC [1]. It has been suggested that visceral pain caused by intraoperative tissue damage during LC is the most significant contributor to pain [2]. Currently, it is recommended to use multimodal analgesia, and the analgesic drugs mainly include opioids; however, opioids increase the risk of adverse events, such as nociceptive hypersensitivity, nausea, vomiting, and mild and short‐term postoperative cognitive dysfunction. Strong opioid analgesics can effectively relieve incisional pain, but when using opioids alone, visceral pain occurs in 12%–20% of patients after laparoscopy [3]. Therefore, it is essential to find lower opioid analgesic regimens to mitigate postoperative pain and improve the quality of anesthesia.

Nalbuphine is an opioid receptor agonist–antagonist with a rapid onset of action (5–10 min) and a long duration of action (3–6 h). Nalbuphine has an agonistic effect on *κ* receptors and an antagonistic effect on *μ* receptors. It can bind to *μ*, *κ*, and *δ* opioid receptors at the same time; thus, it has sedative, analgesic, and respiration inhibitory effects, but its respiration inhibitory effect is minor compared with morphine. The analgesic effect of nalbuphine is comparable to that of morphine, and its addictiveness is significantly weaker than that of morphine. *κ* receptors are distributed in visceral organs, and intravenous administration of nalbuphine can activate *κ* receptors to reduce visceral pain and antagonize *μ* receptors at the same time, offering satisfactory sedative and analgesic effects with a low risk of adverse events [4]. NMDA receptor antagonists are also one of the important components of the perioperative multimodal analgesic protocol, and it has been shown that ketamine exerts its analgesic effects in the dorsal horn of the spinal cord through the NMDA receptor. Ketamine has significant analgesic effects on both visceral and somatic pain and has little effect on the respiratory cycle. In contrast, esketamine is a novel isomer with a higher affinity for NMDA receptors and a lower risk of side effects compared with ketamine [5, 6], and it can effectively decrease the risk of postoperative neurocognitive dysfunction by inhibiting the microglial TLR4/NF‐κB signaling pathway in the central nervous system [7]. However, there are limited data on the effect and dosing of nalbuphine combined with esketamine for lower opioid anesthesia in analgesia (patient‐controlled intravenous analgesia [PCIA]) after LC. In this study, we measured the analgesic effects of nalbuphine combined with esketamine after LC and explored the optimal analgesic combination, thereby providing a reference for the reasonable use of postoperative analgesia for these patients.

## 2. Methods

### 2.1. Study Design

This randomized controlled trial employed a single‐blind design. Specifically, patients were blinded to group allocation, while the anesthesiologists responsible for drug administration (due to involvement in drug preparation) and the principal study designers remained unblinded. However, postoperative data collectors, such as researchers assessing pain scores, sedation levels, and cognitive function, as well as statisticians performing data analysis, were blinded to group assignment to minimize outcome assessment bias. The analgesic pumps were all of a uniform external type.

### 2.2. Ethics

Ethical approval for this study (approval number: Fu Women and Children’s Ethical Approval No. 2024 (2)) was provided by the Ethics Committee of Fuyang Women and Children’s Hospital (Chairperson Luo Chun) on February 7, 2024. The study adhered to the ethical standards outlined in the Declaration of Helsinki and the International Ethical Guidelines for Research Involving Human Health. Patients provided informed consent after receiving comprehensive study details.

### 2.3. Statistical Analysis

Statistical analysis was conducted using SPSS Version 23.0. Measurement data, tested for normality, are presented as mean ± standard deviation (($\overset{¯}{x}$ ± *s*). A one‐way analysis of variance (ANOVA) was employed for between‐group comparisons, while a two‐way repeated‐measures ANOVA was utilized for analyzing multitemporal observations. Quantitative data with a nonnormal distribution are represented as M (P25, P75). The Mann–Whitney *U* test compared two groups, while the Kruskal–Wallis H test was employed for comparisons involving more than two groups. Count data are presented as numbers with percentages, and comparisons were made using the *χ* ^2^ test or Fisher’s exact test. A *p* value of less than 0.05 was deemed statistically significant.

### 2.4. Participants

Ninety patients who underwent elective LC under general anesthesia in Fuyang Women’s and Children’s Hospital and the First People’s Hospital of Hefei (Binhu Hospital District) between February and July 2024 were randomly assigned into three groups: N, NE1, and NE2, with 30 patients in each group. Informed consent was obtained from all participants prior to their inclusion.

Inclusion criteria included the following: (1) aged 18–65 years, with no gender restrictions; (2) American Society of Anesthesiologists (ASA) Health Status Classification Class I–II; (3) body mass index (BMI): 18.5–23.9 kg m^−2^; and (4) willingness to participate and sign informed consent.

The exclusion criteria included the following: (1) comorbid psychiatric and neuropsychological disorders, such as schizophrenia, mood disorders, anxiety disorders, and epilepsy; (2) people with communication impairments; (3) pregnant and breastfeeding women; (4) receiving narcotic analgesics within 6 months; (5) known allergy to the study medication; (6) complicated infections or acute stage of cholecystitis; (7) serious diseases of the liver, kidneys, heart, lungs, etc.; (8) patients with hematologic diseases, including leukemia, aplastic anemia, and pernicious lymphoma; (9) patients with severe hematologic diseases, such as dysplastic anemia, malignant lymphoma, and myelodysplastic syndromes; and (10) patients with other diseases, including angina pectoris, malignant hypertension, and elevated intracranial pressure.

### 2.5. Randomization and Blinding

Researchers collected comprehensive data on patients’ physical and demographic attributes, such as gender, age, height, weight, and education level, during the preoperative visit. The patients and their families were informed about the experimental protocol. Written informed consent was obtained from the patients and their legal guardians. Participants were randomly assigned using SPSS 26.0 software (IBM SPSS, Armonk, NY, USA) into three groups in a 1:1:1 ratio: the N group (nalbuphine 2 mg kg^−1^ with 100 mL of 0.9% sodium chloride solution), the NE_1_ group (esketamine 0.5 mg kg^−1^ and nalbuphine 2 mg kg^−1^ with 100 mL of 0.9% sodium chloride solution), and the NE_2_ group (esketamine 1 mg kg^−1^ and nalbuphine 2 mg kg^−1^ with 100 mL of 0.9% sodium chloride solution). The anesthetist completed the randomization process and treatment allocation the morning before surgery and prepared the medications based on patients’ groups on the day of surgery. The visual analog scale was utilized to evaluate postoperative pain. The group assignments were concealed from patients, data collectors, and follow‐up evaluators. The anesthetist, nurse anesthetist, and researchers involved in patient recruitment were informed of the group assignments.

### 2.6. Anesthesia and Perioperative Analgesia

Patients fasted from solid foods for 8 h and liquids for 4 h. Upon entering the operating room, peripheral veins in the upper limbs were accessed, continuous oxygen was supplied at 2.0–3.0 L/min, and monitoring included electrocardiograms (ECGs), noninvasive blood pressure (BP), heart rate (HR), peripheral capillary oxygen saturation (SpO_2_), and bispectral index (BIS).

Anesthesia was induced sequentially by slow intravenous injection of dexamethasone 10 mg (Jiangsu Hengrui Pharmaceutical Co., Ltd., Lianyungang; batch no.: 10061434), sufentanil 0.4 μg kg^−1^ (Yichang Renfu Pharmaceutical Co., Ltd., Yichang; batch no.: 31A100911), midazolam 0.04 mg kg^−1^ (Jiangsu Enhua Pharmaceutical Co., Ltd., Jiangsu; batch no.: TMD23J01), etomidate 0.3 mg/kg (Jiangsu Enhua Pharmaceutical Co., Ltd., Jiangsu; batch no.: TYTH5), cisatracurium 0.10 mg kg^−1^ (Jiangsu Hengrui Pharmaceutical Co., Ltd., Lianyungang; batch no.: 230729Bl) or rocuronium bromide 0.6 mg kg^−1^ (Jiangsu Hengrui Pharmaceutical Co., Ltd., Lianyungang; batch no.: 139230905). BIS was ≤ 60 and maintained for 5 s, and then a laryngeal mask was inserted or an endotracheal tube was connected to an anesthesia machine for mechanical ventilation. Tidal volume (VT) was set at 6–8 mL kg^−1^, with a respiratory rate (RR) of 14–16 breaths per minute, an inspiratory–expiratory ratio (IR) of 1:1.5, and end‐tidal carbon dioxide partial pressure (*P*_ET_CO_2_) maintained at 35–45 mmHg.

Intraoperative anesthesia was sustained through intravenous administration of propofol at a dosage of 4–8 mg kg^−1^ h^−1^ (Sichuan Guorui Pharmaceutical Co., Ltd., Leshan; batch no.: 23062912) and remifentanil at a dosage of 0.1–0.2 μg kg^−1^ min^−1^, supplied by Yichang Renfu Pharmaceutical Co., Ltd., Yichang (batch no.: 30A09321). The BIS level was maintained between 40 and 60. Intraoperative blood pressure was maintained within 20% of the baseline value. Dopamine at 2 mg was administered for hypotension, while urapidil​ at 10 mg was used for hypertension. Atropine 0.3 mg times^−1^ minute^−1^ was applied when the heart rate was less than 50 beats/minute, and esmolol 10 mg times^−1^ minute^−1^ was administered when the heart rate was higher than 100 beats per minute. The administration of propofol and remifentanil ceased upon completion of suturing, followed by the initiation of postoperative intravenous analgesia using a pump from Jiangsu Aipeng Medical Technology Co., Ltd., Nantong (batch no.: Z01035676) was connected, and the endotracheal tube was removed after natural awakening.

### 2.7. Postoperative Analgesic Dosing and Grouping

Postoperative PCIA analgesia dosing and grouping: Group N received nalbuphine 2 mg kg^−1^ (Yichang Renfu Pharmaceutical Co., Ltd, Yichang; batch no.: 31J100211) + 100 mL of 0.9% sodium chloride solution (Jiangxi Kelun Pharmaceutical Co., Ltd, Fuzhou; batch no.: D24110807). Group NE_1_ received nalbuphine 2 mg kg^−1^ + esketamine 0.5 mg kg^−1^ (Jiangsu Hengrui Pharmaceutical Co., Ltd, Lianyungang; batch no.: 240629BP) + 100 mL of 0.9% sodium chloride solution. Group NE_2_ received nalbuphine 2 mg kg^−1^ + esketamine 1 mg kg^−1^ + 100 mL of 0.9% sodium chloride solution. The background infusion rate of the analgesia pump was 2 mL h^−1^, the first volume was 2 mL, the self‐control infusion volume was 2 mL, and the locking time was 15 min. When the postoperative VAS pain score was > 4, the first choice was to press the analgesic pump once. Intravenouslornoxicam​ 4 mg was used to enhance analgesia when the pump was ineffective.

### 2.8. Outcome Assessments

The following data were collected:

1. General information, including gender, age, height, BMI, operation time, anesthesia time, and pneumoperitoneum pressure (5 min from the beginning of the surgery), etc.
2. VAS pain scores (0: no pain; 1–3: mild pain; 4–6: moderate pain; 7–9: severe pain; 10: worst pain) at rest and during activity (coughing) at 6, 12, 24, and 48 h postoperatively.
3. The total and effective analgesic pump presses, along with the count of patients requiring additional analgesia, were recorded at 6, 12, 24, and 48 h postsurgery.
4. The preoperative Montreal Cognitive Assessment (MoCA) scale scores and those at 6, 12, 24, and 48 h following surgery, which took approximately 10 min to complete. One point was added to patients with education ≤ 12 years. The total score was 30 points, and the normal value was ≥ 26 points.
5. Postoperative Ramsay sedation scores at 6 h, 12 h, 24 h, and 48 h (1, irritable; 2, quiet and cooperative; 3, lethargic, able to follow instructions; 4, sleepy but arousable; 5, sleepy, somewhat responsive to strong stimuli but unresponsive; 6, deeply sleepy, unable to be awakened by calling out; 2–4, satisfactory sedation; 5–6, over‐sedation).

### 2.9. Sample Size Calculation

The study determined the sample size based on the VAS score 24 h postoperatively, with equal allocation across the three groups. Based on preliminary experiments and relevant literature, the mean VAS score in Group N was estimated to be 3.5 ± 1.2. A reduction of 0.8 points in Groups NE1 and NE2 compared to Group N was defined as a clinically meaningful difference. Using *α* = 0.05 (two‐sided) and *β* = 0.20 (80% power), a sample size calculation for ANOVA was performed with PASS 2021 software, which indicated that at least 29 patients per group were required. To account for an estimated dropout rate of approximately 10%, 33 patients per group were planned for enrollment, resulting in a total planned sample size of 99. During actual recruitment, to cover the planned sample size and buffer anticipated exclusions (e.g., screening failures or dropouts), a total of 102 patients were screened, which included the 99 planned patients plus approximately a 3% buffer. Ultimately, 90 patients (30 per group) were successfully enrolled, meeting the statistical power requirements for ANOVA.

## 3. Results

### 3.1. Patient Enrollment

**FIGURE 1:**
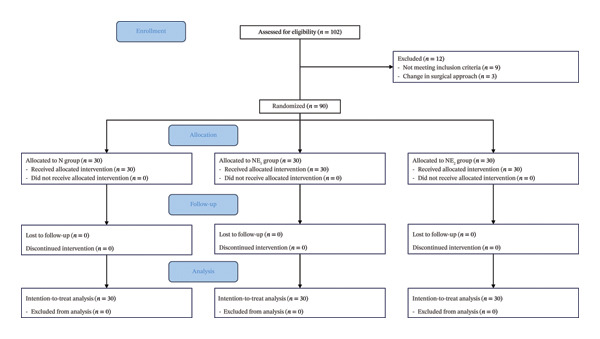
Patient enrollment process.

Summing up, 102 participants were recruited for this study. Ninety patients were enrolled in the study after excluding nine who did not meet the inclusion criteria and three who altered their surgical approach midway. The study involved 90 patients who were randomly divided into three groups, each consisting of 30 patients (Figure 1).

The N group included patients who received nalbuphine 2 mg kg^−1^ and 0.9% sodium chloride solution 100 mL. The NE_1_ group included patients who received nalbuphine 2 mg kg^−1^, esketamine 0.5 mg kg^−1^, and 0.9% sodium chloride solution 100 mL. The NE_2_ group included patients who received nalbuphine 2 mg kg^−1^, esketamine 1 mg kg^−1^, and 0.9% sodium chloride solution 100 mL.

### 3.2. Comparison of General Conditions/Demographic Characteristics

The three groups showed no significant differences in age, gender, BMI, ASA classification, operation time, anesthesia time, or pneumoperitoneum pressure (*p* ≫ 0.05) (Table 1).

**TABLE 1:** Comparison of general conditions/demographic characteristics of the three groups of patients.

Variables	Group N (*n* = 30)	Group NE_1_ (*n* = 30)	Group NE_2_ (*n* = 30)	*F/* *χ* ^2^	*p*
Age (years)	39.60 ± 11.17	46.42 ± 12.74	43.37 ± 13.38	2.319	0.104
Sex (m f^−1^)	14/16	15/15	16/14	0.267	0.875
BMI (kg m^−2^)	21.00 ± 1.53	20.95 ± 2.07	22.46 ± 1.47	2.521	0.099
ASAI/II (examples)	13/17	12/18	14/16	0.271	0.873
Surgery time (min)	47.90 ± 13.08	51.41 ± 14.00	48.44 ± 12.87	0.665	0.517
Pneumoperitoneum pressure (mmHg)	10.85 ± 1.03	11.29 ± 1.55	10.75 ± 1.10	1.674	0.193

*Note:* Data are expressed as mean ± standard deviation.

### 3.3. Comparison of VAS Scores and Ramsay Sedation Scores at Different Time Points

VAS activity and resting pain scores were considerably less in Groups NE_1_ and NE_2_ than in Group N at 6, 12, and 24 h postoperatively (*p* < 0.05). At 48 h postoperatively, the postactivity VAS scores were considerably reduced in both Group NE_1_ and Group NE_2_ (*p* < 0.05). Compared with Group N, Group NE_1_, and Group NE_2_ were not significantly different in terms of resting VAS scores (*p* > 0.05). The NE_2_ group exhibited considerably lower VAS activity pain scores at both 12 and 24 h postoperatively compared to the NE_1_ group (*p* < 0.05) (Table 2).

**TABLE 2:** Comparison of pain scores and Ramsay sedation scores at different time points among the three groups of patients.

		VAS rest	VAS activity	Ramsay
Group N (*n* = 30)	Postoperative 6 h	2.30 ± 0.99	3.62 ± 1.43	1.70 ± 0.54
	Postoperative 12 h	2.20 ± 0.81	3.07 ± 1.33	1.63 ± 0.56
	Postoperative 24 h	1.93 ± 0.55	2.83 ± 1.15	1.87 ± 1.90
	Postoperative 48 h	1.59 ± 0.66	2.33 ± 0.38	2.03 ± 0.96

Group NE_1_ (*n* = 30)	Postoperative 6 h	1.53 ± 0.62^a^	2.37 ± 0.93^a^	1.97 ± 0.32^a^
	Postoperative 12 h	1.42 ± 0.67^a^	1.90 ± 0.66^a^	2.00 ± 0.26^a^
	Postoperative 24 h	1.57 ± 0.37^a^	1.42 ± 0.58^a^	2.16 ± 0.89^a^
	Postoperative 48 h	1.14 ± 0.10^a^	1.16 ± 0.12^a^	2.08 ± 0.50

Group NE_2_ (*n* = 30)	Postoperative 6 h	1.47 ± 0.60^a^	2.50 ± 0.89^a^	2.43 ± 0.57^a^ ^,^ ^b^
	Postoperative 12 h	1.20 ± 0.61^a^	1.70 ± 0.65^a^ ^,^ ^b^	2.30 ± 0.47^a^ ^,^ ^b^
	Postoperative 24 h	1.48 ± 0.61^a^	1.17 ± 0.39^a^ ^,^ ^b^	2.23 ± 0.43^a^
	Postoperative 48 h	1.10 ± 0.38^a^	1.14 ± 0.28^a^	2.07 ± 0.73

Overall comparison	HF coefficient	1.0031	0.8309	0.7345
Intergroup comparison	F, *P*	42.234,0.000	82.941,0.000	9.524,0.000
Intragroup comparison	*F*, *P*	20.038,0.000	42.827,0.000	0.324,0.723
Interaction	*F*, *P*	4.950,0.000	0.764,0.550	1.319,0.264

*Note:* Data are presented as mean ± standard deviation. Compared with Group *N*.

^a^ *p* < 0.05; compared with Group NE_1_.

^b^ *p* < 0.05; VAS: visual analog scale method; Ramsay: sedation score.

Ramsay sedation scores at 6, 12, and 24 h postoperatively were considerably higher in Groups NE_1_ and NE_2_ compared to Group N (*p* < 0.05). Ramsay sedation scores were considerably higher in Group NE_2_ than in Group NE_1_ at both 6 and 12 h postoperatively (*p* < 0.05) (Table 2).

Two‐factor repeated‐measures ANOVA with sphericity correction via the HF coefficient method was used for overall comparisons. Latitude comparisons between groups were performed using LSD‐t tests.

### 3.4. Comparison of the total number of analgesic pump compressions and the number of effective compressions at different time points

The total number of presses and the number of effective presses of the analgesic pumps in the NE_1_ and NE_2_ groups were considerably less than those in the N group (*p* < 0.05). In the NE_2_ group, both the total and effective number of presses were considerably lower than those in the NE_1_ group during the 24 h and 48 h postoperative periods (*p* < 0.05) (Table 3).

**TABLE 3:** Comparison of total number of presses and effective number of presses with analgesic pumps at different time points in the three groups of patients.

	Time point	Total number of compressions	Effective number of compressions
Group N (*n* = 30)	Postoperative 6 h	0.47 ± 0.20	0.23 ± 0.10
	Postoperative 12 h	0.63 ± 0.27	0.43 ± 0.18
	Postoperative 24 h	0.73 ± 0.31	0.57 ± 0.24
	Postoperative 48 h	0.78 ± 0.36	0.57 ± 0.21

Group NE_1_ (*n* = 30)	Postoperative 6 h	0.13 ± 0.05^a^	0.10 ± 0.04^a^
	Postoperative 12 h	0.19 ± 0.08^a^	0.16 ± 0.07^a^
	Postoperative 24 h	0.26 ± 0.14^a^	0.20 ± 0.08^a^
	Postoperative 48 h	0.26 ± 0.15^a^	0.20 ± 0.07^a^

Group NE_2_ (*n* = 30)	Postoperative 6 h	0.10 ± 0.04^a^ ^,^ ^b^	0.09 ± 0.04^a^
	Postoperative 12 h	0.16 ± 0.07^a^	0.13 ± 0.05^a^
	Postoperative 24 h	0.19 ± 0.08^a^ ^,^ ^b^	0.16 ± 0.06^a^ ^,^ ^b^
	Postoperative 48 h	0.19 ± 0.08^a^ ^,^ ^b^	0.16 ± 0.06^a^ ^,^ ^b^

Overall comparison	HF coefficient	0.9064	0.9050
Intergroup comparison	F, *P*	242.768, 0.000	284.925, 0.000
Intragroup comparison	F, *P*	17.452, 0.000	38.826, 0.000
Interaction	F, *P*	2.310, 0.045	9.808, 0.000

*Note:* Data are presented as mean ± standard deviation. Compared with Group *N*. Overall comparisons utilized a two‐factor repeated‐measures ANOVA with sphericity correction, employing the HF coefficient method. Fine comparisons between groups on latitude were performed using LSD‐t tests.

^a^ *p* < 0.05; compared with Group NE_1_.

^b^ *p* < 0.05.

## 4. Comparison of Montreal Cognitive Scores at Different Time Points

**FIGURE 2:**
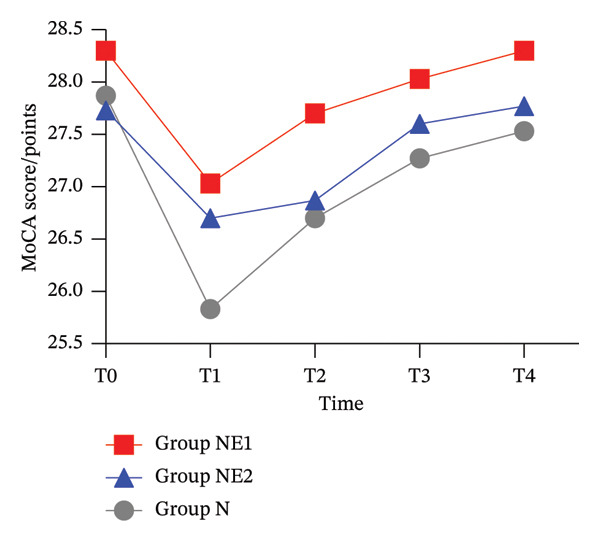
Comparison of Montreal Cognitive Assessment scores at different time points among the three groups of patients. MoCA score: Montreal Cognitive Assessment scale; T0: preoperative; T1: 6 h postoperative; T2: 12 h postoperative; T3: 24 h postoperative; T4: 48 h postoperative.

Comparing the changes in MoCA scale scores between preoperative and postoperative 48 h time points, the difference between the three groups was not statistically significant (*p* > 0.05). Compared to the N group, cognitive scores based on the MoCA scale were significantly higher in both the NE_1_ and NE_2_ groups at 6 h, 12 h, and 24 h postoperative time points (*p* < 0.05) (Figure 2).

### 4.1. Comparison of Adverse Events and Remedial Analgesia Between the Three Groups Within 48 h After Surgery

During the 48 h postoperative period, the NE_1_ and NE_2_ groups experienced a substantially lower incidence of vomiting compared to the N group (*p* < 0.05). The NE_2_ group exhibited a substantially higher rate of drowsiness within 48 h postoperatively compared to the N and NE_1_ groups (*p* < 0.05) (Table 4).

**TABLE 4:** Comparison of adverse events and remedial analgesia in the postoperative 48‐h period.

Variables	Group N (*n* = 30)	Group NE_1_ (*n* = 30)	Group NE_2_ (*n* = 30)	*χ*2	*p*
Vomiting	10 (33)	3 (10)^a^	4 (13)^a^	6.237	0.044
Dizziness	5 (17)	1 (3)	1 (3)	4.957	0.084
Urinary retention	4 (13)	1 (3)	1 (3)	4.957	0.084
Hiccups	1 (3)	0 (0)	1 (3)	1.023	0.600
Respiratory depression	0 (0)	0 (0)	0 (0)	1.023	0.600
Hallucination	0 (0)	0 (0)	0 (0)	1.023	0.600
Pruritus	0 (0)	0 (0)	0 (0)	1.023	0.600
Somnolence	1 (3)	2 (3)	8 (3)^a^ ^,^ ^b^	0.907	0.012
Rescue analgesia	2 (7)	0 (0)	0 (0)	0.821	0.444

*Note:* Data are presented as mean ± standard deviation.

^a^ *p* < 0.05 compared with Group N.

^b^ *p* < 0.05 compared with Group NE_1_.

## 5. Discussion

Pain is usually an unavoidable complication after surgery. Research indicates that over 80% of surgical patients experience acute postoperative pain, with nearly 75% of them reporting pain severity of moderate or higher. Laparoscopic surgery accounts for a sizable percentage of patients with severe pain scores in the early postoperative period after abdominal surgery [3, 8]. Pain causes great suffering to patients and is a major contributor to many postoperative complications affecting cognitive function, patient satisfaction, and delayed recovery [9]. Effective relief of postoperative pain while reducing adverse events is a hot research topic for clinical anesthesiologists. Pain after LC mainly consists of abdominal wall poking pain, visceral pain, and pneumoperitoneum pressure–induced shoulder pain. The abdominal wall incision is infiltrated by the physician, and ultrasound‐guided abdominal wall nerve block has achieved good results; thus, managing to deal with visceral pain after LC is particularly important. PCIA is easy to take care of and has a low risk of infection; thus, it is widely used for postoperative analgesia during the perioperative period. Currently, opioid analgesia is widely used in clinical settings, but it can lead to side effects, such as depressed respiration, nausea and vomiting (N/V), addiction, and nociceptive allergy. Multimodal analgesia can exert analgesic effects while reducing the side effects of various analgesics through multitarget effects. Depending on the mechanism of pain after LC, a satisfactory analgesic effect can be achieved by a combination of two or more drugs, which is the main reason why multimodal analgesia is recommended by ERAS. In this study, we used different doses of esketamine combined with nalbuphine for opioid‐sparing postoperative analgesia dosing through continuous analgesia after PCIA to strengthen analgesic effects, improve the quality of analgesia, reduce the opioid dosage and adverse effects, and mitigate postoperative complications.

Nalbuphine [10] is a mixed opioid receptor agonist–antagonist whose analgesic effect is comparable to that of morphine. *κ* receptor agonists at the spinal cord level can alleviate visceral pain, somatic pain, and referred pain and have a greater analgesic effect in visceral pain caused by abdominal and obstetric surgeries compared with sufentanil. Unlike opioids that activate μ receptors, nalbuphine is a μ receptor antagonist that does not cause addiction, itching, and other adverse effects and reduces the risk of respiratory depression. Due to abnormal respiratory function and impaired metabolic function, patients with malnutrition and obesity are more prone to respiratory depression when using sedative drugs. Therefore, this study used a normal BMI range (18.5–23.9 kg m^−2^) as the inclusion criterion. Despite controversies, the range of 18.5–23.9 kg m^−2^ is widely applicable to the general adult population (aged 18–65 years). Esketamine is the dextrose form of ketamine, which mainly exerts analgesic effects by selectively blocking the nociceptive afferent signals of the reticular bundle of the spinal cord, blocking the transmission of nociceptive signals to the thalamus and cortical areas. It also directly binds to some of the receptors in the center and the dorsal horn of the spinal cord to exert analgesic effects [11, 12]. The clinical use of large amounts of opioids or poor analgesia may lead to central sensitization, which is mainly triggered by C fibers. When the body is subjected to strong and prolonged stimulation, it will activate NMDA receptors in the dorsal horn of the spinal cord to cause central sensitization. By virtue of being an NMDA receptor antagonist, esketamine curbs the onset of central sensitization and diminishes the likelihood of secondary pain phenomena. Furthermore, administration of low‐dose esketamine has been demonstrated to ameliorate both nociceptive hypersensitivity at the surgical site and remifentanil‐associated respiratory depression [3, 13, 14].

The base dose of nalbuphine (2 mg kg^−1^) selected in this study was mainly based on the following two considerations: (1) Literature basis: Multiple studies on analgesia after laparoscopic surgery have shown that nalbuphine can provide effective analgesia when administered as a single intravenous injection or as a background infusion at a dose of 0.1–0.3 mg kg^−1^ [15]. Considering that this study aimed to observe the sustained effect of its combined application with esketamine, we selected a relatively high starting total dose within this range for exploration. (2) Previous practice: In the clinical practice of our hospital, nalbuphine within this dose range has been proven to have good safety and preliminary analgesic effects for similar surgeries. Combining with the synergistic effect of esketamine, we aimed to verify whether this combined dose regimen could achieve better analgesic effects and less total consumption of opioids. The 0.5 mg kg^−1^ and 1 mg kg^−1^ doses of esketamine were selected based on the literature [16] and pretest results. The results of this study showed that compared to Group N, the VAS activity and resting pain scores of Group NE_1_ and Group NE_2_ were substantially less at postoperative 6 h, 12 h, and 24 h. Compared to Group N, the postactivity VAS scores of Group NE_1_ and Group NE_2_ were lower at the 48‐h postoperative time point, suggesting that different dosages of esketamine combined with nalbuphine can offer effective analgesia after LC, and suggesting that the combination of esketamine with nalbuphine can produce a synergistic effect and a more complete analgesic effect. Compared to the NE_1_ group, the VAS activity pain score was reduced in the NE_2_ group at 12 h and 24 h postoperatively, but the differences between the groups did not reach statistical significance at postoperative 48 h. The NE_1_ and NE_2_ groups exhibited significantly fewer total and effective analgesic pump presses compared to the N group. The NE_2_ group exhibited fewer total and effective presses at 24 and 48 h postoperatively compared to the NE_1_ group, indicating that a higher dose of esketamine enhances early postoperative analgesia. This also reflects the superior analgesic effect with the addition of esketamine, which is consistent with the pharmacological effects of nalbuphine and esketamine.

The findings of this study demonstrate that the Ramsay sedation scores were higher in the NE_1_ and NE_2_ groups at 6 h, 12 h, and 24 h postoperatively compared to the N group. The Ramsay sedation scores were also higher in the NE_2_ group at 6 h and 12 h postoperatively compared with the NE_1_ group, suggesting that esketamine combined with nalbuphine can make patients more comfortable. Meanwhile, the Ramsay sedation score increased with an increase in the dose of esketamine, evidenced by an increased incidence of early oversedation and drowsiness. This finding suggests that a high dose of esketamine may increase its side effects, probably due to the synergistic effects with the sedative effects of nalbuphine.

Higher postoperative pain scores have been associated with an increased risk of delirium. In addition, the use of opioids, especially long‐acting opioids, has been linked to an increased risk of postoperative delirium [17]. However, the pathophysiological mechanisms of delirium remain unknown, and neuroinflammation remains a major area of research. Esketamine has been shown to inhibit microglial TLR4/NF‐κB signaling in the central nervous system, ameliorating stress‐induced neuroinflammatory responses and alleviating postoperative neurocognitive dysfunction [7]. This intervention alleviates trauma‐induced chronic neuroinflammation, counteracts blood–brain barrier disruption, mitigates oxidative stress, and suppresses microglial activation [18, 19]. In the present study, the addition of esketamine in both groups significantly increased MoCA scale scores at 6 h, 12 h, and 24 h postoperatively compared with the preoperative level, improving early cognitive function. Since the sympathomimetic effect of esketamine can increase cerebral blood flow with little effect on respiration, it may improve postoperative cognition in elderly patients [20, 21]. The results of this study are consistent with the results of Zhao et al. [22] and Hou et al. [23]. In the 48‐h postoperative period, the incidence of vomiting in the NE_1_ and NE_2_ groups was substantially less than that in the N group. No significant difference was observed in the incidence of other adverse events, which may be due to the fact that the sedative effect of nalbuphine used in combination with the two drugs can decrease the side effects of the psychotropic class of esketamine [24, 25].

In this study, the changes in stress factors in vivo and their effect on the incidence of chronic pain were not detected when using esketamine combined with nalbuphine as postoperative analgesia, which needs further studies. There is a certain subjective variability in the assessment of the VAS pain score, which can be improved by using the pain assessment index.

In conclusion, PCIA with the combination of esketamine and nalbuphine given to patients after LC can effectively relieve postoperative pain in patients with LC, provide good sedation, reduce the use of opioids, and improve cognitive function, with fewer adverse effects. Nalbuphine 2 mg/kg combined with esketamine 0.5 mg/kg for opioid‐sparing anesthesia is a suitable candidate for analgesia after LC, which is safe, effective, and reliable. The use of this strategy should be popularized in clinics.

## Author Contributions

Throughout the research process, Mengyun Zhang was involved in conceptualization, design, data collection, analysis, interpretation, and writing. Hao Wu and Han Zheng assisted in data collection, and Jing Zhang was responsible for collecting other data. Heng Yang and Hong Luo played an important role in the conceptualization, design, interpretation, and manuscript revision of the study. In addition, Heng Yang and Hong Luo established a random assignment sequence to recruit participants and assign the intervention to them. Jiaxuan Wang played an important role in manuscript revision. All authors participated in reviewing the final manuscript for publication.

## Funding

This study was funded by the Hefei City, the seventh cycle of clinical key cultivation speciality construction project (Hewei medical secret​ [2023] 72). Anhui Provincial Health Research Project (Grant No. AHWJ2024Aa20018). Hefei Municipal Healthcare Science and Technology Project (Grant No. Hwk2025zd001).

## Disclosure

This manuscript is not being considered for publication in any other journal. All authors have approved the final version of the manuscript for publication, and necessary approvals from the responsible institutional authorities have been obtained.

## Conflicts of Interest

The authors declare no conflicts of interest.

## Data Availability

The data supporting the findings of this study are available from the corresponding author upon reasonable request.
